# Tailored Treatment Strategies in First Line Therapy for Ovarian Cancer Patients: A Critical Review of the Literature

**DOI:** 10.3390/ph17060778

**Published:** 2024-06-14

**Authors:** Daniela Luvero, Roberto Angioli, Federica Celoro, Francesco Plotti, Corrado Terranova, Federica Guzzo, Gianna Barbara Cundari, Federico Liparulo, Camilla Verdone, Roberto Montera

**Affiliations:** 1 Department of Gynecology, Fondazione Policlinico Universitario Campus Bio Medico, Via Alvaro del Portillo 200, 00128 Roma, Italy; d.luvero@policlinicocampus.it (D.L.); r.angioli@policlinicocampus.it (R.A.); f.plotti@policlinicocampus.it (F.P.); c.terranova@policlinicocampus.it (C.T.); f.guzzo@policlinicocampus.it (F.G.); r.montera@policlinicocampus.it (R.M.); 2 Research Unit of Gynecology, Department of Medicine and Surgery, Università Campus Bio Medico, Via Alvaro del Portillo 21, 00128 Roma, Italy; g.cundari@unicampus.it (G.B.C.); federico.liparulo@unicampus.it (F.L.); camilla.verdone@unicampus.it (C.V.)

**Keywords:** ovarian cancer, PARP inhibitors, maintenance therapy, bevacizumab

## Abstract

Background: Ovarian cancer (OC) is a significant cause of cancer-related mortality in women globally, with a five-year survival rate of approximately 49%. Standard therapy involves cytoreductive surgery followed by chemotherapy. Its poor prognosis has driven interest in alternative therapies such as targeted molecular agents like bevacizumab and poly (ADP-ribose) polymerase inhibitors (PARPi). Materials and Methods: This review systematically searched PubMed from January 2018 to December 2023 for studies on PARPi in OC. Emphasis was on identifying relevant Phase III trials, extracting data on study design, patient demographics, and outcomes. Special focus was on assessing PARPi efficacy, safety, impact on quality of life, and ongoing trials, including those on Clinicaltrials.gov. Results: The efficacy of PARPi in first-line therapy for OC has been extensively studied. Trials like SOLO-1, PRIMA, and ATHENA-MONO have demonstrated significant improvements in progression-free survival (PFS) and overall survival (OS), particularly in patients with BRCA mutations. Additionally, the combination of PARPi with other agents like bevacizumab has shown promising results in extending PFS. However, PARPi treatment is associated with various adverse effects, including hematologic toxicities like anemia, thrombocytopenia, and neutropenia. While most adverse events are manageable, some patients may require dose adjustments or discontinuation of treatment. Importantly, PARPi maintenance therapy has not adversely affected health-related quality of life (HRQoL), with studies reporting similar HRQoL scores between PARPi-treated and placebo-treated patients. Conclusions: PARPi offer effective treatment with manageable side effects, suitable even for medically fragile patients. Individualized dosing can optimize benefits while minimizing adverse events. Exploring diverse treatment approaches, particularly in patients with limited life expectancy or high disease burden, could improve outcomes. Ongoing research is investigating alternative therapies and combinations to broaden treatment options. Combining bevacizumab with PARPi may be justified for first-line and recurrent maintenance therapy. Regardless of mutational status, PARPi should be considered for maintenance therapy in newly diagnosed advanced OC. Platinum sensitivity remains crucial for treatment decisions and predicting survival outcomes.

## 1. Introduction

Ovarian cancer (OC) includes a large spectrum of histopathologic varieties, in which epithelial ovarian cancer (EOC) represents nearly 90% of malignant ovarian neoplasms [1,2,3,4]. Globally, OC ranks sixth among malignant tumors and fifth as a cause of cancer-related mortality in women [5]. Despite the progress, the five-year survival rate is still around 49%, with early-stage cases and specific histologic subtypes exhibiting longer survival [6,7,8,9]. Unfortunately, due to the absence of specific early symptoms, characteristic peritoneal spread, and the absence of effective screening, 75% of patients are diagnosed in the advanced stage [7].

Standard therapy involves surgical staging cytoreduction (residual tumor = 0, R0) followed by platinum-and-taxane-based chemotherapy [10,11,12,13]. Before 2010, treatment advancements predominantly centered on factors such as the administration route, dose intensity (e.g., intraperitoneal chemotherapy), dose scheduling (e.g., weekly schedule, dose-dense paclitaxel), and substitutions of cytotoxic chemotherapy due to mechanisms of resistance or toxicities (e.g., pegylated liposomal doxorubicin [PLD] instead of taxane) [14].

Recurrent EOC, inevitable for most women, carries a poor prognosis, prompting multiple lines of chemotherapy with unsatisfactory outcomes due to drug-resistant cancer clones. For these reasons the scientific interest in alternative drugs or maintenance for first-line approaches has intensified [15].

Trials integrate targeted molecular therapies, including bevacizumab, poly (ADP-ribose) polymerase inhibitors (PARPi), and immunotherapy have been developed [16].

Since 2011, anti-angiogenetic target therapies have shown activity in association with standard chemotherapy in several trials; bevacizumab (Bev) in two Phase III trials particularly showed an increase in progression-free survival (PFS) and overall survival (OS) in patients with newly diagnosed [16,17,18,19], platinum-sensitive relapsed (PSR) [20,21,22], and platinum-resistant subsetting [23].

More recently, PARPi have emerged as significant therapeutic options in ovarian cancer. Three PARPi: olaparib—niraparib, and rucaparib—were approved in 2018 for recurrent ovarian cancer settings and later in first-line settings, but only two of them (olaparib, niraparib) are currently used in clinical practice as maintenance therapy in patients with OC.

PARPi exploit the compromised DNA repair function through the homologous recombination (HR) pathway. This mechanism is particularly relevant in cells with mutations in HR repair genes, such as BRCA1 and BRCA2. Essentially, PARPi capitalize on this weakened DNA repair capability, leading to the accumulation of DNA damage and eventual cell death, particularly in cancer cells with impaired HR repair mechanisms. This therapeutic approach illustrates the concept of synthetic lethality, where the combination of PARP inhibition and defective HR repair pathways induces cell death, offering a targeted strategy for treating cancers with compromised DNA repair mechanisms [24].

PARPi exhibit activity not only in cancer cells with a homologous recombination deficiency (HRD) like BRCA mutation but also in patients with a proficiency profile [24].

Thanks to great improvements in terms of outcome in recurrence settings, the therapeutic landscape is evolving, with a shift from using PARPi in relapsed disease to their integration in first-line maintenance therapy. Recent Phase III studies emphasize the optimal benefits of initiating PARPi at the beginning of treatment, challenging the exclusive reservation for disease relapse scenarios [25,26,27,28].

Our review aims to evaluate the efficacy and main side effects of PARP inhibitors in first line setting, their impact on patients’ quality of life, and provide a critical analysis of ongoing studies. 

## 2. Materials and Methods

This review was initiated by conducting comprehensive literature searches on PubMed, encompassing papers published from January 2018 up to December 2023. The search utilized specific terms related to PARPi in ovarian cancer, including “olaparib”, “niraparib”, “rucaparib”, and “first-line maintenance therapy in ovarian cancer”. 

The focus was primarily on identifying relevant Phase III studies within the specified timeframe.

Relevant data from identified studies were extracted and categorized based on study design, patient characteristics, intervention details, and key outcomes. Emphasis was placed on obtaining information regarding the efficacy and toxicity of PARPi, their impact on the quality of life of patients, and any ongoing trials that could contribute to the evolving landscape of OC treatment.

Ongoing trials related to OC and PARPi were explored on Clinicaltrials.gov. The research specifically utilized filters for “Ovarian Cancer”, “PARP inhibitors”, and “Phase III”. 

Information from these trials was scrutinized for potential insights into emerging therapeutic approaches and the future direction of PARPi research in OC.

## 3. Results

### 3.1. PARPi Efficacy in First Line Setting 

The effectiveness of PARPi has been extensively demonstrated by previous studies, highlighting overall survival (OS) and progression free survival (PFS) that justify their use as first-line therapy for maintenance in high-grade OC [25,26,27,28,29,30,31,32,33,34,35,36,37,38] (Table 1).

In 2018, the SOLO-1 trial randomized 391 patients with BRCA1 and/or BRCA2 mutation, FIGO Stage III or IV high-grade serous or endometrioid ovarian/primary peritoneal/fallopian tube cancer, regardless of surgical outcome and/or complete/partial response after first-line platinum-based chemotherapy. With a median follow-up of 41 months, olaparib demonstrated a 70% reduction in the risk of disease progression or death compared to placebo (Kaplan–Meier estimate of the 3-year rate of freedom from disease progression and death, 60% vs. 27%; HR: 0.30; 95% CI: 0.23–0.41; *p* < 0.001) [25].

Recently, a 7-year descriptive analysis reported a clinically meaningful OS advantage in patients treated with olaparib vs. placebo (67% vs. 46.5% respectively). In addition, according to Kaplan-Meier estimates, after a 7-year follow-up, 45.3% of olaparib patients and 20.6% of placebo patients survived without having received a first subsequent treatment [29].

In 2019, the PRIMA trial, 733 patients were randomized, and 150 of them (around 20%) did not have a BRCA mutation. The inclusion criteria for this study mirrored those of the PRIMA trial, with the notable exception that patients without BRCA mutations were not excluded. Consequently, the study was broadened to encompass the entire population with advanced-stage cancer, regardless of mutational status. Additionally, the selected patients had a high risk of progression, meaning those with Stage III disease and no residual macroscopic disease after upfront surgery were excluded. Among those with HRD (50.9%), niraparib significantly improved PFS compared to placebo, both in patients with BRCA mutations (22.1 months vs. 10.9 months; HR: 0.40) and those without (19.6 months vs. 8.2 months; HR: 0.50). In patients with homologous recombination proficiency (HRp), niraparib also prolonged PFS compared to placebo (8.1 months vs. 5.4 months; HR: 0.68), suggesting additional mechanisms of action beyond DNA damage repair [26].

In the ongoing ATHENA-MONO trial, as of the data cutoff analysis in 2022, the random assignment included 427 patients to rucaparib and 111 patients to placebo, with a breakdown of 185 versus 49 in the HRD population. The inclusion criteria were FIGO Stage III or IV high-grade serous or endometrioid ovarian, primary peritoneal, or fallopian tube cancer, regardless of surgical outcome and/or complete or partial response after first-line platinum-based chemotherapy, irrespective of biomarker status and postoperative residual disease status. In the HRD population, the median PFS (95% CI) reached 28.7 months (23.0 to not reached) with rucaparib, 11.3 months (9.1 to 22.1) with placebo (HR: 0.47; 95% CI: 0.31–0.72; *p* = 5.0004). For the HRD-negative population, the median PFS periods were 12.1 months (11.1 to 17.7) with rucaparib and 9.1 months (4.0 to 12.2) with placebo (HR: 0.65; 95% CI: 0.45–0.95, *p* < 0.001) [27]. To date, these statistically significant findings support Rucaparib’s efficacy in extending PFS among both HRD-positive and HRD-negative patients, although with a lesser degree of effect in the latter group.

The strategic use of biologic combinations targeting various aberrant pathways in OC represents a promising treatment approach for high-grade serous ovarian cancer (HGSC), considering its intricate genomic landscape. Specifically, PARPi combinations hold potential to induce heightened DNA damage and trigger increased HRD.

The early investigation into PARPi combinations concentrated on the integration of antiangiogenic agents. A pivotal Phase I trial scrutinized the pairing of olaparib with the oral VEGFR inhibitor cediranib, thereby establishing the optimum dosage for Phase II investigations [30]. The cediranib/olaparib combination exhibited enhanced median PFS compared to olaparib monotherapy in platinum-sensitive HGSC, prompting the initiation of pivotal Phase III trials [31]. Furthermore, olaparib has been synergistically paired with bevacizumab, demonstrating promising tolerability and is presently undergoing rigorous evaluation in Phase III trials [32].

The exploration of combining antiangiogenic agents with PARPi stemmed from the hypothesis that antiangiogenics could sensitize BRCAwt tumor cells to PARPi by inducing hypoxia [30]. Phase II trials showed improved PFS with olaparib and cediranib combinations in platinum-sensitive recurrent ovarian cancer (ROC) patients (17.7 vs. 9.0 mos, HR: 0.42; 95% CI: 0.23–0.76; *p* = 0.005) (NCT01116648) [31]. Additionally, niraparib combined with bevacizumab demonstrated enhanced PFS in platinum-sensitive ROC patients (median PFS was 11.6 months) (NCT02354131) [32]. These promising results have led to ongoing Phase III trials assessing the efficacy of PARP inhibitors alone or in combination with antiangiogenics in ovarian cancer patients (NCT02502266, NCT02446600) [31].

The advantage of adding olaparib maintenance to bevacizumab following platinum-based chemotherapy in first-line OC was shown in the PAOLA-1 trial. This study enrolled 806 patients. The inclusion criteria were FIGO Stage III or IV high-grade serous or endometrioid ovarian, primary peritoneal, or fallopian tube cancer with no evidence of disease and complete or partial response after platinum-based chemotherapy plus bevacizumab regardless of biomarker status or clinical risk. Following a median follow-up of 22.9 months, the median PFS was higher with olaparib plus bevacizumab compared to placebo plus bevacizumab regardless of the patient’s genetic profile. In HRD patients, the HR (olaparib group vs. placebo group) for disease progression or death was 0.33 (95% CI: 0.25–0.45, *p* < 0.001), corresponding to a median PFS of 37.2 months in the olaparib group versus 17.7 months in the placebo group. For patients with HRD-positive tumors without BRCA mutations, the HR was 0.43 (95% CI: 0.28–0.66, *p* < 0.001), resulting in a median PFS of 28.1 months in the olaparib group compared to 16.6 months in the placebo group [28].

In 2022, the PRIME trial confirmed the efficacy of using a PARPi as maintenance therapy in first-line settings regardless of the mutational state and postoperative residual disease status. This study was conducted in China and randomized 384 patients. By data cutoff (30 September 2021), median follow-up for PFS was 27.5 (IQR, 24.7–30.4) months. Patients treated with niraparib experienced a significantly extended median PFS compared to those who received placebo (24.8 vs. 8.3 months; HR: 0.45; 95% CI: 0.34–0.60; *p* < 0.001. Subgroup analyses consistently revealed treatment benefits in both the HRD-positive population (HR: 0.48; 95% CI: 0.34–0.68, *p* < 0.001) and HR-positive (HR: 0.41, 95% CI: 0.22–0.75, *p* < 0.001) subgroups [33,34].

In Table 1, the Phase III trials in first line setting are summarized.

### 3.2. PARPi Side Effects 

The efficacy of PARPi has been thoroughly established, and likewise, the side effects and adverse effects of these drugs have been thoroughly investigated (Table 2).

In the SOLO-1 trial, during the course of the clinical trial and within the 30-day period following the discontinuation of the intervention, the most common adverse events (AEs) (all grades) were nausea, fatigue or asthenia, and vomiting [25]. Nausea was the most common AE in the first month of maintenance olaparib; however, its prevalence and severity decreased rapidly. AEs were predominantly Grade 1–2, apart from anemia, which was the most common Grade ≥ 3 AE. The highest occurrence of anemia was noted at the 6-month mark, demonstrating a subsequent decline in the prevalence of Grade 2 or more severe cases over time. The occurrence of neutropenia and thrombocytopenia remained minimal, predominantly Grade 1 for thrombocytopenia and predominantly Grade 2 or higher for neutropenia. Most patients in the olaparib group experiencing anemia, neutropenia, or thrombocytopenia had their adverse events resolve or improve. Conversely, in the placebo group, the incidence of hematologic adverse events remained low throughout the observation period.

No clinically significant changes from baseline in clinical chemistry parameters (including albumin, alanine aminotransferase, aspartate aminotransferase, alkaline phosphatase, gamma glutamyltransferase and bilirubin) occurred in the olaparib or placebo groups. Elevated blood creatinine levels were identified as an AE in 8.1% of patients receiving olaparib and in 1.5% of patients in the placebo group. Notably, all instances of increased blood creatinine were categorized as Grade 1, and none led to the discontinuation of the study drug [35].

Fortunately, no AEs occurring during the trial or within 30 days after intervention discontinuation resulted in fatalities [25].

Regarding specific outcomes, acute myeloid leukemia manifested in 1% of patients in the olaparib group (3 out of 260 patients) and was absent in the placebo group (0 out of 130 patients). All three instances of acute myeloid leukemia occurred more than 30 days after the conclusion of treatment with olaparib.

Regarding extra-hematologic adverse events, incidences of new primary cancers were reported in 2% of the olaparib group and 2% of the placebo group. Cases of pneumonitis or interstitial lung disease were reported in 2% of the olaparib group and none of the placebo group [25].

Lastly, additional symptoms that emerged included diarrhea, constipation, dysgeusia, arthralgia, abdominal pain, headache, dizziness, decreased appetite, upper abdominal pain, dyspepsia, cough, back pain, and dyspnea.

Management of AEs typically involved measures such as dose interruption or reduction, with discontinuation being a less frequent course of action. Overall, AEs led to dose interruption in 51.9% of olaparib patients versus 16.9% of placebo patients; dose reduction in 28.5% versus 3.1%, respectively; and study drug discontinuation in 11.5% versus 2.3%, respectively, as listed in Table 3 [32]. Nausea and anemia were identified as the primary adverse events leading to discontinuation [25].

Following a 7-year follow-up, the safety profile of maintenance olaparib remained consistent with previous data. The most frequently reported AEs of any grade in olaparib-treated patients included nausea, fatigue/asthenia, vomiting, and anemia, with anemia being the most common Grade ≥ 3 AE. Serious AEs were observed in 21.2% of olaparib patients and 13.8% of placebo patients. Since the primary data cutoff on 17 May 2018, one new case of myelodysplastic syndrome (MDS) was reported in the olaparib group (0.4%), and one case of acute myelomonocytic leukemia (AML) was reported in the placebo group (0.8%). Over the 7-year follow-up, four cases of MDS/AML were reported in the olaparib group (1.5%), and one was reported in the placebo group (0.8%). New primary malignancies were documented in 14 olaparib patients (5.4%) and 8 placebo patients (6.2%) over the 7-year period, with 6 new cases in olaparib patients (2.3%) and 3 in placebo patients (2.3%) since the 5 March 2020 data cutoff [29].

In the PRIMA trial, among patients receiving niraparib, the most prevalent grade 3 or higher AEs included anemia (31.0%), thrombocytopenia (28.7%), and neutropenia (12.8%). Dose reductions were implemented in 70.9% of the patients in the niraparib group. The incidence of treatment discontinuation due to adverse events was 12.0% for niraparib compared to 2.5% for placebo (Table 3). Myelosuppressive AEs, primarily thrombocytopenia (4.3% in the niraparib group), were the primary cause for discontinuation. A single case of MDS was identified in one patient from the niraparib group. Low-grade nausea and fatigue were commonly reported in both groups. Importantly, no deaths related to niraparib treatment were documented throughout the trial [26].

Prolonged monotherapy displayed a low rate of discontinuations due to AEs. Introduction of individualized starting doses (ISD) improved safety, reducing the proportions of patients with Grade ≥ 3 treatment-emergent AEs (62.7% vs. 78.4%) and treatment-related Grade ≥ 3 AEs (53.8% vs. 73.0%) compared to fixed starting doses (FSD) [36].

In PRIME, adverse event rates were generally lower compared with PRIMA, potentially because all PRIME patients received an ISD. [39] Most treatment-emergent adverse events (TEAEs) were well controlled, which led to low treatment discontinuation rates in both groups (niraparib (6.7%) vs. placebo (5.4%)) [34].

In the ATHENA-MONO trial, the most prevalent AEs, reported in over 40% of patients in either group, included nausea, asthenia/fatigue, anemia, and increased ALT/AST. Grade ≥ 3 AEs were more frequent in the rucaparib group (60.5%) than the placebo group (22.7%), with anemia and neutropenia being the most common in the rucaparib group. Hypertension was the primary grade ≥ 3 AE in the placebo group. Elevated ALT/AST events were mostly Grade 1 or 2, normalizing during treatment without signs of liver injury. Minor AEs were rash, insomnia, myalgia, or an increase in blood creatinine (11.1%) MDS and AML were reported in two rucaparib patients (0.2%), and none were reported in the placebo group. Treatment interruption due to AEs occurred more frequently in the rucaparib group (60.7%), along with dose reduction (49.4%) and discontinuation (11.8%), commonly due to anemia [27].

In the PAOLA-1 trial, fatigue, nausea, and anemia were more frequent in those receiving olaparib plus bevacizumab, while hypertension was more common in the placebo group. Serious AEs occurred in 31% of patients in both trial groups, with anemia more prevalent in the olaparib plus bevacizumab group and hypertension more common in the placebo plus bevacizumab group. Fatal events occurred in <1% of olaparib patients and 1% of placebo patients. MDS, AML, or aplastic anemia were reported in 1% of olaparib plus bevacizumab patients and <1% of placebo plus bevacizumab patients. New primary cancers occurred in 1% of olaparib patients and <1% of placebo patients. Grade 1 or 2 pneumonitis, interstitial lung disease, or bronchiolitis were observed in 1% of olaparib patients, and none were observed in the placebo group. AEs were primarily managed through dose modification rather than discontinuation, with anemia and nausea being the most common reasons for olaparib discontinuation (Table 3) [28].

Patients undergoing maintenance therapy with olaparib plus bevacizumab exhibited a higher likelihood of experiencing AEs commonly associated with bevacizumab, such as hypertension (43% vs. 4%). Notably, the highest incidence of hypertension was observed in the bevacizumab-alone group (55%) [38].

### 3.3. Quality of Life (QoL) in Phase III Trials 

The PFS benefit observed with PARPi maintenance monotherapy and combination therapy in newly diagnosed advanced OC patients didn’t adversely affect health-related quality of life (HRQoL). This was supported by patient-centered outcomes like quality-adjusted PFS (QA-PFS) and time without significant symptoms of toxicity (TWiST) or quality-adjusted TWiST (Q-TWiST), which consider the adverse effects of PARPi.

In studies like SOLO1, PRIMA, and ATHENA-MONO, maintenance therapy with PARPi showed no clinically significant difference in HRQoL scores compared to placebo [25,26,27,28]. PARPi maintenance monotherapy in SOLO1 and PRIMA was associated with significant improvements in QA-PFS and TWiST or Q-TWiST [26,39,40,41]. Similarly, in PAOLA-1, no clinically meaningful difference in HRQoL was observed between maintenance olaparib plus bevacizumab and placebo plus bevacizumab, although olaparib plus bevacizumab showed significant gains in TWiST over placebo plus bevacizumab [28,39,40,41].

## 4. Discussion and Future View

PARPi have revolutionized the long-term management of OC by significantly improving OS and PFS, a milestone unparalleled by any therapy since the introduction of platinum-based treatments in the 1900s. However, it is imperative to underscore that the foremost prognostic determinant remains surgical intervention and the pursuit of radicality. The attainment of minimal residual disease through increasingly advanced surgical approaches has markedly enhanced the prognosis of OC patients. Nevertheless, challenges persist regardless of surgical proficiency, emphasizing the importance of addressing patients subjected to suboptimal cytoreduction (i.e., with non-zero residual disease). Such patients, especially those without BRCA mutations, continue to represent the most prognostically disadvantaged cohort. Consequently, the studies under scrutiny, featuring disparate inclusion criteria (including BRCA mutation status and presence/absence of residual disease), should not be juxtaposed in attempts to delineate drug efficacy or superiority. Rather, their synthesis underscores the recognition of the potential beneficial effect of PARPi even in patients with a poorer prognosis, as demonstrated by the PRIMA and PRIME trials. This stands in contrast to the SOLO1 trial, which focused on a theoretically more manageable patient subset with significantly better prognosis, albeit in entirely theoretical terms.

PARPi have demonstrated both efficacy in terms of outcome and a safe toxicities profile. Nevertheless, these adverse effects should not dissuade us from regarding them as a therapeutic option, even in medically fragile patients undergoing disease and chemotherapy. It has been established that tailoring the dosage for each patient based on factors such as overall health, comorbidities, body weight, and platelet count can optimize benefits and minimize adverse events, thereby preserving the individual’s quality of life.

Although the validity of these studies has been established, there are still no guidelines driving us towards one therapeutic approach over another. Nevertheless, gynecologic oncology is constantly evolving and is already exploring alternative scenarios to PARPi or alternative combinations to expand therapeutic options while ensuring efficacy [42,43,44].

## 5. Conclusions

In conclusion, considering literature data some PARPi (i.e., Niraparib) can be used as maintenance therapy in patients with newly diagnosed advanced stage OC regardless of mutational state. 

Bevacizumab has demonstrated significant therapeutic benefit in terms of PFS especially in the recurrent setting and in those patients with more severe disease which may benefit from receiving bevacizumab, adding to neoadjuvant chemotherapy (NACT) increasing the intraoperative resection rate.

Furthermore, bevacizumab has been demonstrated to be effective in improving clinical outcomes in patients with advanced-stage ovarian tumors undergoing radical surgery (R0), indicating complete removal of the primary tumor. This suggests that adding bevacizumab to standard therapy could lead to better survival and disease control in HRD-positive patients undergoing radical surgery and first-line PARPi treatment. Bevacizumab, in fact, may enhance the effectiveness of PARPi in OC treatment: their combined use could have a synergistic effect in combating tumor growth and spread, potentially offering greater benefits in HRD-positive patients with R0-operated tumors compared to single-agent treatment.

Surely, in both cases (use of PARPi and of bevacizumab) platinum sensitivity can be still considered, and this is currently the main issue in the treatment decision path and one of the most crucial factors for predictive survival outcomes. In fact, the response to chemotherapy, along with the Kelim index and the trend of biomarkers, allows us to assess the patient’s platinum sensitivity and consequently her prognosis, providing insight into the most suitable maintenance therapy. Understanding the patient’s prognosis enables us to select the most appropriate drug: for patients with an excellent response to interval debulking surgery (IDS) and chemotherapy, I might consider administering a PARPi rather than an angiogenesis inhibitor, reserving the latter for cases with less favorable prognoses.

The maintenance therapies analyzed so far have generally been well tolerated by patients, although not without side effects. Therefore, the current aim is to tailor therapy for each patient based on their specific comorbidities. For instance, in patients with neutropenia or thrombocytopenia, it is preferable to avoid PARPi, while in those with hypertension, bevacizumab is not particularly recommended. This concept holds particularly true when referring to niraparib, as studies focusing on this drug have differentiated between ISD and FSD based on BMI and platelet count.

To summarize, depending on the stage of the disease, the degree of oncological radicality, and the patient’s health status, it would be wise to explore various therapeutic regimens rather than relying solely on a single-drug approach, considering the specific characteristics of each patient. In patients with limited life expectancy and a high load of disease (ascites, pleuric effusion, dyspnea, etc.), in which we can benefit from quick results regarding the symptoms, enhancing the efficacy of PARPi with a neoangiogenesis inhibitor could considerably improve quality of life and avoid progressive therapeutic approaches. Despite the increased risk of adverse effects, personalized and carefully calibrated therapy tailored to each patient’s needs offers superior efficacy compared to monotherapy.

## Figures and Tables

**Table 1:** Phase III trials in first line setting (PARPi alone vs. PARPi + beva).

Authors	Study Name	Patients	Target Population	Administered Drug	Primary EP	Secondary EP
Moore 2018 [25]	SOLO-1 NCT01844986	N = 391 Olaparib 260/391; Placebo 131/391	Newly diagnosed, stage III–IV, BRCAm, CR/PR after platinum-based chemotherapy, regardless of clinical risk	Olaparib tablets ^1^ (300 mg twice daily) vs. placebo	PFS	Second PFS, OS, time from randomization to the first subsequent therapy or death, time from randomization to the second subsequent therapy or death, and health-related quality of life
González-Martín 2019 [26]	PRIMA NCT02655016	N = 733 Niraparib 487/733; Placebo 246/733	Newly diagnosed, stage III–IV, CR/PR after platinum-based chemotherapy, regardless of biomarker status, higher clinical risk	Niraparib ^2,3^ 300 mg once daily FSD, or 200 or 300 mg once daily ISD vs. placebo	PFS	Second PFS, OS, time until the first subsequent therapy, pharmacokinetic analyses and patient-reported outcomes
Li 2023 [34]	PRIME NCT0370931	N = 384 Niraparib 255/384; Placebo 129/384	Newly diagnosed, stage III–IV, CR/PR after platinum-based chemotherapy, regardless of biomarker status and postoperative residual disease status	Niraparib ^2^ 300 or 200 mg once daily ISD vs. placebo	BICR-assessed PFS in the ITT population	OS and TFST in the ITT population, PFS and OS in the HRD subgroup
Monk 2022 [27]	ATHENA-MONO NCT03522246	N = 538 Rucaparib 427/538; Placebo 111/538	Newly diagnosed, stage III–IV, CR/PR after platinum-based chemotherapy, regardless of biomarker status and postoperative residual disease status	Rucaparib ^1^ (600 mg twice daily) vs. placebo	PFS per RECIST	OS, ORR, DOR, BICR-assessed PFS per RECIST
Ray-Coquard [28]	PAOLA-1 NCT02477644	N = 806 Ola + Beva 537/806; Placebo + Beva 269/806	Newly diagnosed, stage III–IV, NED/CR/PR after platinum-based chemotherapy plus beva, regardless of biomarker status or clinical risk	Olaparib ^1^ tablets (300 mg twice daily) + beva 4 vs. Placebo + beva ^4^	The time from randomization until investigator-assessed disease progression or death	Time from randomization until second disease progression or death, OS, time until the first subsequent therapy or death, and the global health status–quality of life

Beva: bevacizumab, BICR: blinded independent central review, BRCAm: BRCA1 or BRCA2 mutation, CR: complete response, DOR: duration of response, FSD: fixed starting doses, HRD: homologous recombination deficiency, ISD: individualized starting doses, ITT: intent-to-treat, NED: no evidence of disease, Ola: olaparib, ORR: objective response rate, OS: overall survival, PFS: progression-free survival, PR: partial response, RECIST: response evaluation criteria in solid tumors, TFST: time to first subsequent therapy. ^1^ Capped at 2 years. ^2^ Capped at 3 years. ^3^ Protocol amended to incorporate an ISD of 200 mg once a day for patients with a baseline body weight <77 kg, a platelet count of <150,000/mm^3^, or both. ^4^ 5 mg/kg every 3 weeks for up to 15 months in total.

**Table 2:** PARPi’s side effects.

	OLAPARIB	NIRAPARIB	RUCAPARIB	OLAPARIB + BEVA
Fatigue/Asthenia		++	+++	
Nausea	+++	+++	+++	
Hypertension	+++			+++
Anemia	+++	+++	+++	+++
Lymphopenia	+++			
Arthralgia	++		+	++
Vomiting	++	++	++	
Abdominal pain	++	++	++	
Diarrhea	++		++	++
Neutropenia	++	++	+++	+
Leukopenia	++			+
Urinary tract infection	++			+
Headache	+	++	+	
Constipation	+	++	+	
Thrombocytopenia	+	+++	++	+
Proteinuria	+			+
Increased ALT/AST	+		+++	+
Insomnia		++	+	
Blood creatinine increased			+	

BEVA: bevacizumab. The symbol “+” repeated once to three times represents the percentage of patients who developed adverse effects associated with the individual drug, indicating approximately greater than 40%, between 20% and 40%, and less than 20%, respectively.

**Table 3:** PARPi discontinuation, dose reduction, and dose interruption rate.

	SOLO-1	PRIMA	PRIME	ATHENA-MONO	PAOLA-1
Led to discontinuation of intervention	OLAPARIB Any Grade: 30 (12) Grade 3–4: NA PLACEBO Any Grade: 3 (2) Grade 3–4: NA	NIRAPARIB 58 (12) PLACEBO 6 (2.5)	NIRAPARIB 17 (6.7) PLACEBO 7 (5.4)	RUCAPARIB 50 (11.8) PLACEBO 6 (5.5)	OLAPARIB + BEVA Any Grade: 109 (20) Grade ≥ 3: NA PLACEBO + BEVA Any Grade: 15 (6) Grade ≥ 3: NA
Led to dose reduction	OLAPARIB Any Grade: 74 (28) Grade 3–4: NA PLACEBO Any Grade: 4 (3) Grade 3–4: NA	NIRAPARIB 343 (70.9) PLACEBO 20 (8.2)	NIRAPARIB 103 (40.4) PLACEBO 8 (6.2)	RUCAPARIB 210 (49.4) PLACEBO 9 (8.2)	OLAPARIB + BEVA Any Grade: 220 (41) Grade ≥ 3: NA PLACEBO + BEVA Any Grade: 20 (7) Grade ≥ 3: NA
Led to dose interruption	OLAPARIB Any Grade: 135 (52) Grade 3–4: NA PLACEBO Any Grade: 22 (17) Grade 3–4: NA	NIRAPARIB 385 (79.5) PLACEBO 44 (18.0)	NIRAPARIB 160 (62.7) PLACEBO 25 (19.4)	RUCAPARIB 258 (60.7) PLACEBO 22 (20.0)	OLAPARIB + BEVA Any Grade: 291 (54) Grade ≥ 3: NA PLACEBO + BEVA Any Grade: 65 (24) Grade ≥ 3: NA

BEVA: Bevacizumab, NA: not available.
